# Robust rankings: Review of multivariate assessments illustrated by the Shanghai rankings

**DOI:** 10.1007/s11192-014-1313-8

**Published:** 2014-05-06

**Authors:** Leo Freyer

**Affiliations:** Totentanz 14, 4051 Basel, Switzerland

**Keywords:** Objective weighting, Robustness, Fault tolerance, Shanghai ranking, C02, 62H30

## Abstract

Defined errors are entered into data collections in order to test their influence on the reliability of multivariate rankings. Random numbers and real ranking data serve as data origins. In the course of data collection small random errors often lead to a switch in ranking, which can influence the general ranking picture considerably. For stabilisation an objective weighting method is evaluated. The robustness of these rankings is then compared to the original forms. Robust forms of the published Shanghai top 100 rankings are calculated and compared to each other. As a result, the possibilities and restrictions of this type of weighting become recognisable.

## Introduction

The contribution of this study to scientometric research is to demonstrate fault tolerance with multivariate rankings. In this context Shanghai rankings serve merely as a specific example to illustrate the method.

### Error sources

Kendall (1955) writes ‘…what the ranking loses in accuracy it gains in generality, for if we stretch the scale of measurement… the ranking remains unaltered.’ It therefore seems reasonable that inaccurate data would barely influence rankings, which otherwise look reliable.

Robustness in statistics signifies the insensitivity of a result to small deviations from the assumptions (Huber and Ronchetti 2009). Slight data aberrations are considered as deviations from the assumed accuracy. Random measuring errors are inevitable as an expression of natural noise.

With Shanghai rankings (also known as ARWU = Academic Ranking of World Universities) Liu et al. (2005) rely on error rates of <1 % for counting errors and <2 % for attribution errors. Van Raan (2005) has provided evidence for error rates in attribution of approximately 7 % for the methodology used in Shanghai. This high prevalence is thought to be mainly due to the off-label use of scientist’s citation indices as an evaluation method for their universities.

### Weighting types

Ding and Qiu (2011) distinguish between subjective and objective weighting types and have tested different weighting algorithms for university rankings. Every weighting that is solely based on quantitative differences between indicators is considered as objective or evidence-based. Subjective or arbitrary weightings are not fully comprehensible. Analogously, they could be defined as ‘eminence-based’.

Wiesemüller et al. (2003) mention specifically that no weighting is entirely free from subjective influences, if different methodologies are available, for example. Objective weighting is then concerned with minimising subjective influences and making them quantifiable.

Practical usage of objective weighting can be found in the selection of examination questions (Lienert and Raatz 1994) or in the variance principle of insurance theory (Walz 2004).

If the number of test items clearly exceeds the number of available indicators, a weighting method for differentiation becomes essential. While in the first Shanghai ranking of 2003 all indicators were considered to be equal, in subsequent years the influence of two of the six indicators has been limited by some ambiguous weighting (Billaut et al. 2010).

This review is concerned with multivariate assessments. As rankings attach to them, they are influenced in the same way and can serve as an illustration.

## Materials and methods

The methodology of Shanghai rankings has been adopted for direct comparability despite its obvious weaknesses (Billaut et al. 2010). This means that all subjective weights continue to be used. Additional variability weights are introduced. Subjective and objective weights do not conflict with each other.

### The test system

The test system consists of specially developed simulation programs. Some parameters are pre-selectable:

- Either random numbers or real ranking data are available.
- The number of indicators with ranking data is set to 6, whereas it is adjustable with random numbers.
- The length of ranking orders is selectable up to 100.
- The maximum field size is limited by the available computing power to currently 600, e.g. 100 test items × 6 indicators.
- Different weighting algorithms as well as no weighting are available. In each test two of these possibilities are compared by means of the same data.

**Fig. 1:**
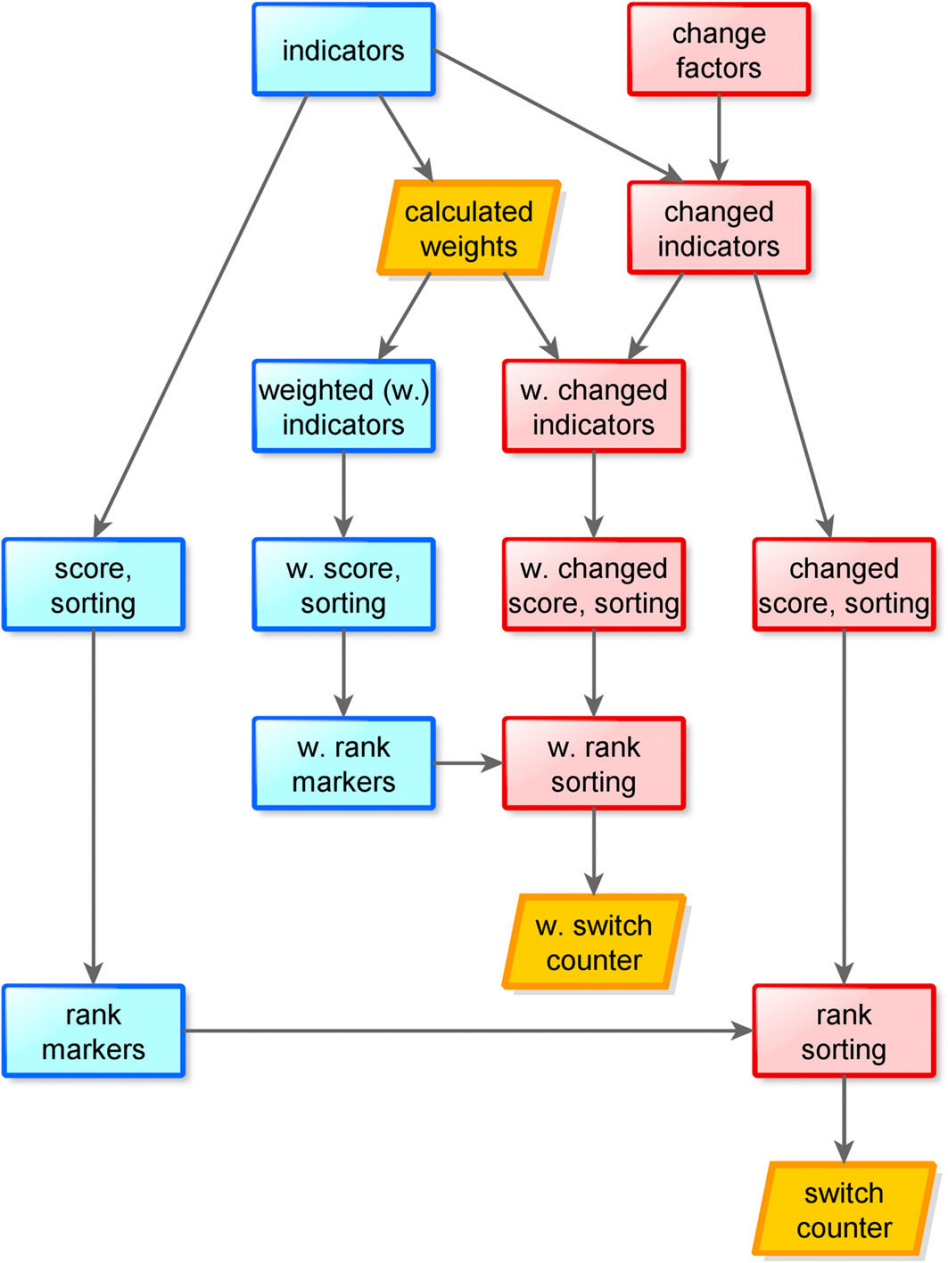
Modular test system for rankings

Figure 1 shows the modular structure of the test system with the inner loop specified for weighting and the outer one for comparison.

### Functioning of the test system

1. Each data line contains the indicators of a test item, i.e. a specific university. The total score of indicators is calculated per line. This leads to an unweighted ranking (1_u_…T_u_).
2. The weight of every indicator is calculated according to a selected weighting algorithm, e.g. a measure of variability.
3. The value of every indicator is multiplied by its calculated weight. The total weighted scores are generated line by line. Altogether this results in a further ranking, which is weighted (1_w_…T_w_).
4. Both rankings are standardised to the same maximum score.
5. Every line is given a specific rank marker for each ranking.
6. In the original data field a pre-set number of randomly chosen points is altered intentionally.
7. With these modified data all calculations are repeated. The previously calculated weights are left unchanged. This leads to two more scores for each line and to two further rankings (1_um_…T_um_, 1_wm_…T_wm_).
8. For the analysis of robustness the original ranking orders are restored by interchanges of neighbouring ranks. The smallest required number of such switching operations is determined (Kendall 1955). The more robust a ranking is, the fewer interchanges are needed to restore the original sequence.
9. In each test run the type of weighting with fewer interchanges receives a point. No point is given for equal switch numbers.
10. This single simulation is repeated many times with different data and varying alterations. The points for each type of weighting are totalled.

### Indicators and attributed weights

The six indicators of Shanghai rankings are currently defined according to Liu et al. (2013).

*Alumni*, weight 10 %: The total number of an institution winning Nobel prizes and Fields medals. Alumni are defined as those who obtain Bachelor’s, Master’s or Doctor’s degrees from the institution. If a person obtains more than one degree from an institution, the institution is considered once only. Different weights are set according to the decades in which the degrees were awarded.

*Award*, weight 20 %: The total number of the staff of an institution winning Nobel prizes in Physics, Chemistry, Medicine and Economics and Fields medals in Mathematics. Staff is defined as those who work at an institution at the time of winning the prize. Different weights are set according to the decades in which the degrees were awarded.

*HiCi*, weight 20 %: The number of Highly Cited Researchers in 21 subject categories. These individuals are the most cited within each category. If a Highly Cited Researcher has two or more affiliations, he/she was asked to estimate his/her weights for each affiliation.

*N&S*, weight 20 %: The number of papers published in Nature and Science within the last 5 years. To distinguish the order of author affiliation, a weight of 100 % is assigned for corresponding author affiliation, 50 % for first author affiliation, 25 % for next author affiliation, and 10 % for other author affiliations.

*PUB*, weight 20 %: Total number of publications indexed in Science Citation Index-Expanded and Social Science Citation Index during the last year. When calculating the total number of papers of an institution, a special weight of two was introduced for papers indexed in Social Science Citation Index.

*PCP* (Per Capita Power), weight 10 %: The weighted scores of the above five indicators divided by the number of full-time equivalent academic staff. If the number of academic staff for institutions of a country cannot be obtained, the weighted scores of the above five indicators is used.

The attributed weights of the six indicators may differ by a factor of two. This uneven treatment is not explained by the producers of the ranking. It is also evident that each indicator contains in its construction further attributed weights which may differ up to one magnitude. With the HiCi indicator possible internal weights are no longer explicitly quantifiable. Thereby this indicator loses its reproducibility, which was in 2005 declared as standard also by Liu et al.

### Computer assistance

The ranking shifts of the individual institutions are calculated with MS Excel^®^. Plotting the results is done with DPlot^®^ from HydeSoft Computing, LLC. Distances and statistical tests are calculated with the universally applicable Mathematica^®^ software package from Wolfram Research, Inc. Special programs have been developed in Mathematica^®^ to simulate fault tolerance with rankings. Anyone who wants to reproduce this kind of software can contact the author in order to obtain support.

## Results

### Weighting principle

**Fig. 2:**
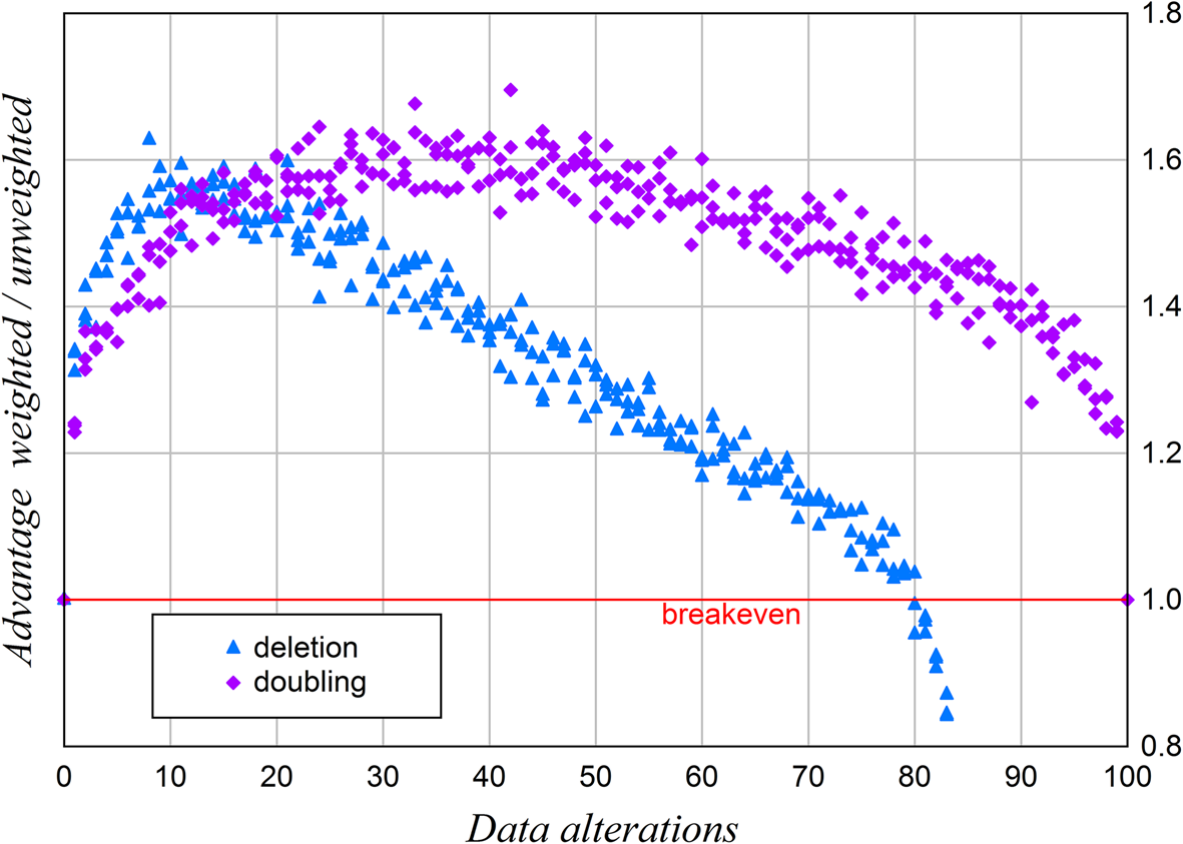
Robustness of rankings with variability weighting. Deletion and doubling. 10 test items, 10 indicators, random data, *n* = 10,000 per data point

In a first simulation uniformly distributed pseudo-random numbers are used to generate 10 test items with 10 artificial indicators each. The standard deviations and means of such indicators differ in small samples only because of real deviations from the uniform distribution. The values of each indicator are weighted by the variation coefficient, which is a normalised, dimensionless variability measure. The variation coefficient is defined as the standard deviation divided by the arithmetic mean. A varying number of points in the data field are either set to zero or their values are doubled. Each test is run 10,000 times per data point. The results are given in Fig. 2.

Figure 2 shows that weighted rankings are more robust over a wide range of data alterations. Variability weighting therefore seems to be a suitable method to obtain robust rankings.

### Upgrading

**Fig. 3:**
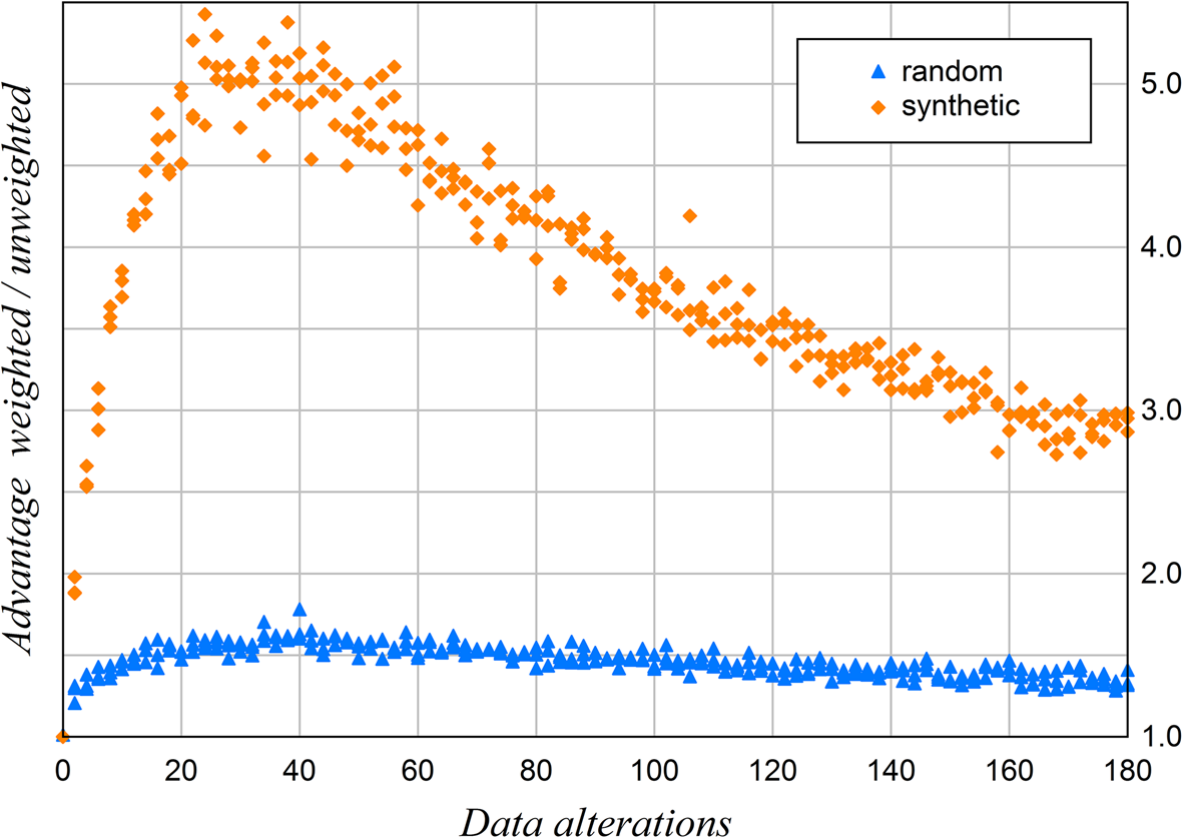
Robustness of rankings with deletion-doubling combination. 30 test items, 6 indicators, random and synthetic ranking data, *n* = 5,000 per data point

The number of test items is tripled while the number of indicators is reduced. Both alteration types are combined: In each test one half are deletions and the other half are doubled values. Synthetic rankings are generated by using Shanghai top 100 data from 2004 to 2012 in a random mix. The number of data alterations is varied at intervals of 2 (Fig. 3).

Figure 3 shows the differing robustness of rankings derived either from random numbers or from real data. Both rankings have undergone the same weighting procedure according to their variation coefficients. If deletion of data and doubled values occur simultaneously, weighted rankings are favourable over the whole range. Rankings made up of Shanghai ranking data become several times more robust through weighting than rankings consisting of random numbers.

### Synthetic rankings

**Fig. 4:**
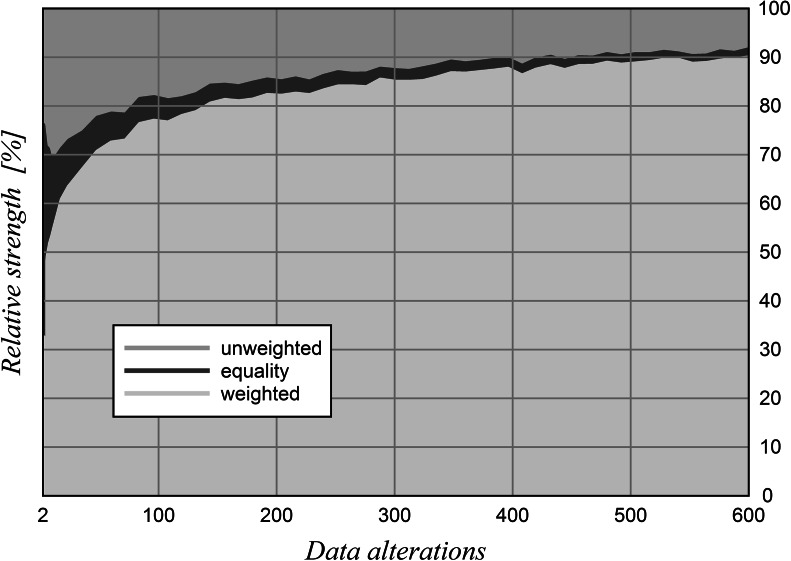
Robustness of rankings with alterations of ± 2 %. 100 test items, 6 indicators, synthetic ranking data, *n* = 5,000.* Key* The segments in *vertical direction* indicate how frequently each particular ranking type performs better

Randomly mixed Shanghai rankings with lengths of 100 are generated, i.e. the data for each ranking position have been randomly chosen from the years 2004 to 2012, which allows for a vast number of varied rank orders to work with. Data alterations are limited to ±2 %. Such minor alterations can be taken as a simulation of counting errors. The awarding of points is further differentiated: cases with equal robustness of both rankings are also taken into account (Fig. 4).

Figure 4 shows that already with slight changes of a small number of data the weighted ranking performs better in the majority of cases. With an increasing number of alterations this relative frequency gradually improves further until a maximum of about 90 %. The number of undecidable cases forms the smallest fraction already with few changes and diminishes further until the possible maximum of 600.

### Shanghai rankings

Table 1 shows the variation coefficients for each indicator. The 150 top ranked universities from every year of Shanghai rankings are taken into account. This table shows clear differences between the indicators and relatively similar data within each individual indicator. As a result some critical aspects are apparent.

**Table 1:** Variation coefficients of indicators for the top 150 universities according to Shanghai rankings

Year	Alumni	Award	HiCi	N&S	PUB	PCP
2003	–	1.152	0.570	0.502	0.248	0.459
2004	0.852	1.104	0.555	0.500	0.241	0.493
2005	0.873	1.108	0.549	0.516	0.244	0.391
2006	0.857	1.085	0.530	0.523	0.241	0.369
2007	0.853	1.074	0.516	0.516	0.248	0.375
2008	0.870	1.062	0.514	0.518	0.242	0.377
2009	0.855	1.069	0.509	0.524	0.241	0.385
2010	0.852	1.056	0.513	0.519	0.243	0.393
2011	0.838	1.024	0.517	0.501	0.245	0.404
2012	0.878	1.041	0.521	0.510	0.241	0.404

*Alumni* bachelors, masters or doctors of an institution winning Nobel prizes and Fields medals, *Award* number of the staff of an institution winning Nobel prizes and Fields medals, *HiCi* highly cited researchers, *N&S* articles published in Nature and Science, *PUB* articles indexed in Science Citation Index-expanded and Social Science Citation Index, *PCP* The weighted scores of the above five indicators divided by the number of full-time equivalent faculty members

#### PCP

Although one could expect that this size-dependent indicator would most likely show consistency, in fact, it scatters most when variation coefficients are compared over the years given in Table 1. The size of an institution has been defined as the number of full-time equivalent faculty members (Liu and Cheng 2007). To acquire these data one is dependent on the current administrations of each university. Such data are difficult to obtain and verify (Florian 2007) and are possibly also politically influenced. The extensive scattering of PCP and likewise its subjectively reduced weight seem to confirm my own experience in getting these data from some Swiss universities.

#### Award

Of the indicators considered, the Award indicator, i.e. Nobel prizes and Fields medals, shows the largest variation coefficient. This finding is quite amazing, if one considers the numerical limitation of prize-givings. Liu et al. (2005) have done a great deal to increase scattering. First of all, they consider the awards over the last 100 years, i.e. over several former generations of research workers. Then the size of the university at the time of the award is used as a multiplier. If it is already difficult to determine the present number of full-time equivalent faculty members of a university, this becomes even more difficult for the period covering the past 100 years. I wonder whether such questions can be answered seriously without the study of sources on site. At most these deductions lead to approximate values, which make the indicator entirely irreproducible. If, in addition, a designated Nobel prize winner changes university, his or her research performance is erroneously attributed to the new institution. The relationship between such number games and the current ‘research performance’ (Liu and Cheng 2007) of institutions remains quite incomprehensible.

#### Alumni

The Alumni indicator has been calculated since 2004 with regard to the university work history of the awarded scientists. In light of the critique by van Raan (2005) and others, the problematic inclusion of size into the indicator has in fact been omitted. Nevertheless, Alumni has a desirably wide range, which furthermore is very stable in the time frame (Table 1). As a result, the Alumni indicator would actually form an ideal substitution for the Award indicator. It remains an open question as to why Alumni is not rated at its full value.

### The inner circle

Those research institutions which were among the Shanghai top 100 every year are currently being examined further. At the end of 2012 this so called inner circle had a size of 83. The inner circle is further divided into research institutions inside and outside the USA.

The official Shanghai rank minus the weighted rank, which has been calculated from the variation coefficients of the same year, leads to an annual ranking shift for each university. The definition shift = rank_unweighted_ − rank_weighted_ has been chosen in the sense that a positive shift coincides with a better ranking in the weighted case.

Table 2 shows the research institutions of the inner circle belonging to the USA in alphabetical order with their medians of ranks and of ranking shift for 2003–2012.

**Table 2:** Selected US research institutions in alphabetical order with their medians of ranks and of ranking shift for 2003–2012

	Institutions of the USA	State	Median of weighted rank	Median of ranking shift	Median of unweighted rank
1	Boston University	Massachusetts	105	−17.5	80.5
2	Brown University	Rhode Island	66.5	0	69.5
3	California Institute of Technology	California	6	−0.5	6
4	Carnegie Mellon University	Pennsylvania	38	20	59
5	Case Western Reserve University	Ohio	76.5	6	80.5
6	Columbia University	New York	9	−1	7.5
7	Cornell University	New York	12	0	12
8	Duke University	North Carolina	55.5	−24	32
9	Harvard University	Massachusetts	1	0	1
10	Massachusetts Institute of Technology (MIT)	Massachusetts	3.5	1	5
11	Michigan State University	Michigan	108.5	−24	84
12	New York University	New York	29	1	30.5
13	Northwestern University	Illinois	34	−3.5	30
14	Pennsylvania State University, University Park	Pennsylvania	69	−26	42.5
15	Princeton University	New Jersey	7	1	7.5
16	Purdue University, West Lafayette	Indiana	67.5	1	68.5
17	Rice University	Texas	67	22	89
18	Rockefeller University	New York	17.5	13.5	31
19	Rutgers, The State University of New Jersey, New Brunswick	New Jersey	51.5	−1	50.5
20	Stanford University	California	5	−2	2
21	The Johns Hopkins University	Maryland	19	0	19
22	The Ohio State University, Columbus	Ohio	92.5	−28	63
23	The University of Texas at Austin	Texas	43	−5	38
24	The University of Texas Southwestern Medical Center at Dallas	Texas	33.5	8.5	40
25	University of Arizona	Arizona	106.5	−30.5	76.5
26	University of California, Berkeley	California	4	0	4
27	University of California, Davis	California	79.5	−36.5	44.5
28	University of California, Irvine	California	45	0.5	46
29	University of California, Los Angeles	California	13	−0.5	13
30	University of California, San Diego	California	14	0	14
31	University of California, San Francisco	California	19	−1.5	18
32	University of California, Santa Barbara	California	30.5	4	34.5
33	University of Chicago	Illinois	8	1	9
34	University of Colorado at Boulder	Colorado	30	3	34
35	University of Florida	Florida	92	−29	62.5
36	University of Illinois at Urbana-Champaign	Illinois	22	3	25
37	University of Maryland, College Park	Maryland	40	−3	37.5
38	University of Michigan, Ann Arbor	Michigan	33	−12	21
39	University of Minnesota, Twin Cities	Minnesota	40.5	−9.5	30.5
40	University of North Carolina at Chapel Hill	North Carolina	68.5	−22.5	46.5
41	University of Pennsylvania	Pennsylvania	15	0	15
42	University of Pittsburgh	Pennsylvania	79	−29	51
43	University of Rochester	New York	76.5	−3.5	74.5
44	University of Southern California	California	51	−3.5	46.5
45	University of Utah	Utah	97.5	−17.5	82.5
46	University of Washington, Seattle	Washington	17.5	−1	16
47	University of Wisconsin, Madison	Wisconsin	16	1.5	17
48	Vanderbilt University	Tennessee	37	5	41.5
49	Washington University in St. Louis	Missouri	27	1	28.5
50	Yale University	Connecticut	11	0	11

Tables 2 and 3 show that higher-ranking institutions generally have smaller ranking shifts. This corresponds with a better alignment of both rankings in the foremost third. The differences averaged for other members of the inner circle can amount to more than 30 ranks.

**Table 3:** Selected research institutions outside the USA in alphabetical order with their medians of ranks and of ranking shift for 2003–2012

	Institutions outside the USA	Country	Median of weighted rank	Median of ranking shift	Median of unweighted rank
1	Karolinska Institute	Sweden	38.5	8	45.5
2	King’s College London	UK	67	9	77
3	Kyoto University	Japan	24	−0.5	23.5
4	Leiden University	Netherlands	67.5	3.5	71.5
5	McGill University	Canada	87	−24	63
6	McMaster University	Canada	83	6.5	89
7	Osaka University	Japan	100	−33	67.5
8	Pierre and Marie Curie University, Paris 6	France	39.5	2	41.5
9	Swiss Federal Institute of Technology Zurich	Switzerland	21	4.5	24.5
10	Technical University Munich	Germany	44.5	10	55
11	The Australian National University	Australia	62.5	−4.5	58
12	The Hebrew University of Jerusalem	Israel	60	4.5	64.5
13	The Imperial College of Science, Technology and Medicine	UK	24	0.5	23.5
14	The University of Edinburgh	UK	59	−6	52
15	The University of Manchester	UK	54	−4.5	46
16	The University of Tokyo	Japan	26	−6.5	20
17	University College London	UK	23	−1.5	21.5
18	University of Basel	Switzerland	74	12.5	86.5
19	University of Bristol	UK	64	−0.5	62
20	University of British Columbia	Canada	41	−5.5	36
21	University of Cambridge	UK	2	2	4
22	University of Copenhagen	Denmark	45.5	4	45.5
23	University of Heidelberg	Germany	58.5	6	64
24	University of Helsinki	Finland	73	0.5	73
25	University of Melbourne	Australia	83.5	−7.5	76.5
26	University of Munich	Germany	51	1.5	52.5
27	University of Oslo	Norway	50	19	68
28	University of Oxford	UK	10	0	10
29	University of Paris Sud (Paris 11)	France	32.5	15	48.5
30	University of Toronto	Canada	30.5	−5.5	24
31	University of Zurich	Switzerland	54	2	56.5
32	Uppsala University	Sweden	53.5	14	66.5
33	Utrecht University	Netherlands	44.5	−0.5	44.5

### US vs. non-US comparison

**Fig. 5:**
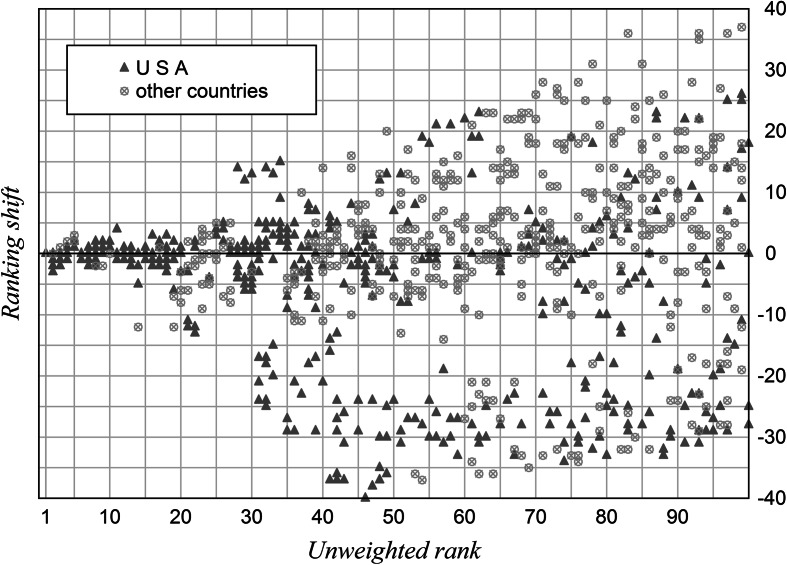
Ranking shift versus rank. The top 100 institutions 2003–2012 with their ranking shifts in relation to original rank

The US institutions seem to score generally weaker in weighted rankings (Tables 2 and 3). For each Shanghai top 100 ranking the shifts of each subgroup–US and non-US institutions—are cumulated and their median is calculated. Both subgroups are compared in all ranking shifts annually by means of a Mann–Whitney test. The differences are expressed quantitatively in Table 4 and shown in Fig. 5.

**Table 4:** The top 100 research institutions with cumulated ranking shifts (Δ) and medians of ranking shifts 2003–2012, differentiated between US and non-US institutions, ranking shifts compared by means of Mann–Whitney tests

Year	Cumulation of US Δ	Median of US Δ	Cumulation of non-US Δ	Median of non-US Δ	*p* (Mann–Whitney)
2003	−312	−1	67	2	0.0038
2004	−267	0	152	2	0.0078
2005	−299	−1	160	2	0.0054
2006	−306	0	106	4	0.0130
2007	−333	−1	107	2	0.0043
2008	−271	−1	88	3	0.0119
2009	−272	−1	71	2	0.0086
2010	−261	0	72	2	0.0081
2011	−248	−1	34	2	0.0084
2012	−223	−1	67	1	0.0313

Table 4 shows that the annual cumulation of ranking shifts is negative for US institutions but positive for non-US institutions. The medians of the US group are negative or zero, whereas they are positive in the other group. The right-hand column gives the probabilities of error (p) of the Mann–Whitney test, assuming that the ranking shifts of both subgroups belong to different populations. As a result, both subgroups are significantly different for each year.

The underlying causes of this decomposition could be of linguistic or historical nature. They are not the subject of this investigation. However, by means of objective weighting the overall picture of a ranking should not change essentially in order to still be classified as robust. Through variability weighting the problematic Award indicator becomes further amplified. Surprisingly, the PUB indicator cannot differentiate the institutions sufficiently and therefore receives only a low weight (Table 1). This weak performance of the PUB indicator is seen in connection with the nonlinear characteristic adjustment (Florian 2007) by the ranking team.

In light of all the inaccuracies of Shanghai rankings I do not intend to publish their explicit weighted forms. The raw data of the top 150 institutions are listed in Freyer (2012) for the purpose of verification.

As a result, variability weighting is not to be understood as a corrective for methodological discrepancies. On the contrary, these inaccuracies can thereby be discovered and analysed.

### Distance measures

Distance comparisons show whether the differences between rankings as a whole are relevant. The similarity of rankings is compared in Table 5 by means of the Damerau–Levenshtein distance (DLD) according to Damerau (1964), as specified in Wolfram Research, Inc (2013): The DLD between two strings u and v gives the number of one-element deletions, insertions, substitutions and transpositions required to transform u to v.

**Table 5:** Comparisons of top 100 ranking data in weighted and unweighted forms

Year (y)	DLD w_y_−u_y_	DLD *u*_y_−*u*_y+1_	First difference	Overlap (%)
2003	82	83	3rd place	90
2004	78	67	2nd place	92
2005	81	55	3rd place	93
2006	79	53	3rd place	89
2007	81	56	2nd place	90
2008	81	51	2nd place	91
2009	80	57	2nd place	92
2010	79	62	2nd place	93
2011	79	57	2nd place	93
2012	81	–	2nd place	94

*Key* Column 2 gives the Damerau-Levenshtein distance (DLD) between weighted and unweighted rankings of the same year; column 3 shows the DLD between the unweighted rankings of consecutive years; column 4 gives the foremost rank, where a modification occurs through weighting; column 5 shows the percentage of congruence between both rankings

A Mann–Whitney test presents a significant difference between the DLD of weighted and unweighted rankings on the one hand and the DLD of unweighted consecutive rankings on the other. The probability of error is 0.004, assuming that the differences from 2003 to 2012 are not random. Consequently the weighted and unweighted rankings of the same year differ significantly more than the unweighted rankings of two consecutive years.

### Mechanism

**Fig. 6:**
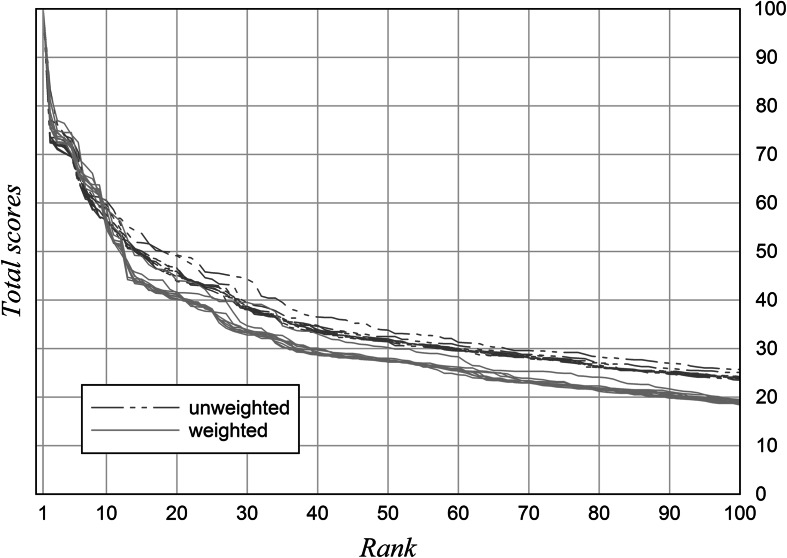
Distribution of total score in relation to rank. Top 100 rankings 2003–2012

Figure 6 shows the total score in relation to rank for the top 100 rankings 2003–2012. As a result, the scale range is better utilised through weighting. This provides an explanation for the robustness of these rankings.

## Discussion

### Problems relating to Shanghai rankings

When I first heard about Shanghai ranking, I sent an e-mail to N.C. Liu. I welcomed his approach, but suspected that a ranking of several hundred items by only five indicators could be inherently unstable. I asked the ranking team whether they had tried different weights for the indicators in order to improve stability. Liu e-mailed back: ‘The weights of the five criteria are rather arbitrary. Changing the weights could change the position of a specific university, however, it did not make much difference on the ranking picture in general’ (Liu 2004, personal communication). Table 4 shows that this statement is neither applicable to the top 100 for 2003 nor for the subsequent years.

Table 5 quantifies these general ranking pictures by means of DLD. While there is a considerable distance between the rankings of 2003 and 2004, the later rankings obviously resemble each other much more. The big difference between 2003 and 2004 is mainly due to the introduction of the Alumni indicator. This can be verified by recalculating the 2004 ranking without Alumni.

Despite the recognised need for improvement and a corresponding announcement (Liu and Cheng 2007), the official ranking methodology has not been developed further since 2004. What happened? The ranking team has primarily been focused on credibility through consistent rankings over the years. Therefore, I would assume that they are fairly busy now with the adjustment of the data using their so-called ‘standard statistical techniques’ against ‘any significant distorting effect’ (Liu and Cheng 2007) whatsoever.

Instead of a linear characteristic, the PUB indicator shows a power function with an exponent <1 (Florian 2007). Such curves intensify small inputs and weaken large ones. In this way an indicator receives different weights according to its size, which has not hitherto been justified in the case of rankings.

The official methodology cannot be developed substantially without some temporary loss of consistency as long as the number of indicators considered is so limited. Even the claim of a mere research ranking (Liu and Cheng 2007) cannot be met by Shanghai rankings. For this purpose indicators from application-oriented research, like for instance the number of patents, should not be entirely absent.

### Methodological limitations

The limitations for variation coefficient weights relate to the different types of error. While the influence of random errors in robust rankings is reduced, this is not the case with systematic errors.

On the other hand, the influence of systematic errors on the calculation of variability weights can be reduced. For this reason the variation coefficient is preferred for weighting. If, for example, all values of an indicator are systematically underestimated by the same percentage, the variation coefficient remains unaffected. Standard deviation and variance, which would also lead to robust rankings, do not offer this advantage.

Another case to be investigated is error propagation within rankings. If, for instance, the size of an institution has been falsely determined, all size-dependent indicators are affected. With reference to Shanghai rankings PCP and the Award indicator would be directly compromised. These subsequent errors are predictable according to the theory of errors. So far the applied programs cannot produce such combined errors for simulations.

Another limitation arises from the type of weighting algorithm used here. Weights for stabilisation should lead to a wider range of total scores, as shown in Fig. 6. A single indicator for differentiation is optimally selective if its values are dispersed uniformly over the whole scale. The extent to which a real indicator resembles this ideal can be termed as its discriminatory power. The usual measures of dispersion like standard deviation do not cover this key feature of indicators satisfactorily. For example, they overestimate peripheral values and they do not account for the shape of the frequency distributions. Defining more sensitive algorithms for quantifying the discriminatory power is feasible.

### Reasons for variability weighting

To evaluate indicators according to their faculty of discrimination is methodologically quite reasonable. On the one hand, the application of variability weighting is justifiable on a practical basis with more reliable results.

On the other hand, a theoretical explanation comes from palaeontology, where the changing spread of variation has been described by Gould (1996) as a crucial element in the process of evolution. From this perspective, rankings appear rather as an element of morphology than as a political instrument. The advancement of ranking methodology in the context of systems science seems to be both probable and desirable.

## Conclusion

Robust rankings in the form shown here are a first approach. New weighting algorithms are easy to evaluate by direct comparisons in the test system.

The extension of objective weighting to other structure-finding procedures (Backhaus et al. 1994) seems reasonable if this leads to better results, for instance to more reliable dendrograms.

Multivariate rankings with subjective weighting or no weighting at all are relatively unstable. Such weak constructions should not be taken as absolute, but only treated with wellfounded caution.

Error-tolerant methods should become routine if a ranking could serve for decision making, and therefore is not an aim in itself. Robust rankings are an attempt to avoid inappropriate evaluations.

In all cases, objective weighting provides a different view on the same data and thereby offers a second opinion: ‘Same same but different’.
